# Botanicals and Oral Stem Cell Mediated Regeneration: A Paradigm Shift from Artificial to Biological Replacement

**DOI:** 10.3390/cells11182792

**Published:** 2022-09-07

**Authors:** Anami Ahuja, Pankaj Kumar Tyagi, Manoj Kumar, Naveen Sharma, Suraj Prakash, Radha, Deepak Chandran, Sangram Dhumal, Nadeem Rais, Surinder Singh, Abhijit Dey, Marisennayya Senapathy, Lejaniya Abdul Kalam Saleena, Arjun Shanavas, Pran Mohankumar, Sureshkumar Rajalingam, Yasodha Murugesan, Marthandan Vishvanathan, Sangeetha Kizhakkumkara Sathyaseelan, Sabareeshwari Viswanathan, Keerthana Krishna Kumar, Suman Natta, Mohamed Mekhemar

**Affiliations:** 1 Department of Biotechnology, Dr. A.P.J. Abdul Kalam Technical University, Lucknow 226031, India; 2 Department of Biotechnology, Meerut Institute of Engineering and Technology, Meerut 250005, India; 3 Department of Biotechnology, Noida Institute of Engineering & Technology, Greater Noida 201306, India; 4 Chemical and Biochemical Processing Division, ICAR–Central Institute for Research on Cotton Technology, Mumbai 400019, India; 5 Division of Biomedical Informatics, Indian Council of Medical Research, New Delhi 110029, India; 6 School of Biological and Environmental Sciences, Shoolini University of Biotechnology and Management Sciences, Solan 173229, India; 7 Department of Veterinary Sciences and Animal Husbandry, Amrita School of Agricultural Sci-ences, Amrita Vishwa Vidyapeetham University, Coimbatore 642109, India; 8 Division of Horticulture, RCSM College of Agriculture, Kolhapur 416004, India; 9 Department of Pharmacy, Bhagwant University, Ajmer 305004, India; 10 Dr. S. S. Bhatnagar University Institute of Chemical Engineering and Technology, Panjab University, Chandigarh 160014, India; 11 Department of Life Sciences, Presidency University, 86/1 College Street, Kolkata 700073, India; 12 Department of Rural Development and Agricultural Extension, College of Agriculture, Wolaita Sodo University, Wolaita Sodo P.O. Box 138, Ethiopia; 13 Department of Food Science and Nutrition, Faculty of Applied Sciences, UCSI University, Kuala Lampur 56000, Malaysia; 14 Division of Medicine, Indian Veterinary Research Institute, Bareilly 243122, India; 15 School of Agriculture and Biosciences, Karunya Institute of Technology and Sciences, Coimbatore 641114, India; 16 Department of Agronomy, Amrita School of Agricultural Sciences, Amrita Vishwa Vidyapeetham University, Coimbatore 642109, India; 17 Department of Seed Science and Technology, Amrita School of Agricultural Sciences, Amrita Vishwa Vidyapeetham University, Coimbatore 642109, India; 18 Department of Plantation Crops and Spices, Kerala Agricultural University, Vellanikkara, Thrissur 680656, India; 19 Department of Soil Science and Agricultural Chemistry, Amrita School of Agricultural Sciences, Amrita Vishwa Vidyapeetham University, Coimbatore 642109, India; 20 ICAR—National Research Centre for Orchids, Pakyong 737106, India; 21 Clinic for Conservative Dentistry and Periodontology, School of Dental Medicine, Chris-tian-Albrecht’s University, 24105 Kiel, Germany

**Keywords:** oral stem cells, mesenchymal cells, osteogenic proliferation, dental, oral cavity, bioactive compounds, PRISMA

## Abstract

Stem cells are a well-known autologous pluripotent cell source, having excellent potential to develop into specialized cells, such as brain, skin, and bone marrow cells. The oral cavity is reported to be a rich source of multiple types of oral stem cells, including the dental pulp, mucosal soft tissues, periodontal ligament, and apical papilla. Oral stem cells were useful for both the regeneration of soft tissue components in the dental pulp and mineralized structure regeneration, such as bone or dentin, and can be a viable substitute for traditionally used bone marrow stem cells. In recent years, several studies have reported that plant extracts or compounds promoted the proliferation, differentiation, and survival of different oral stem cells. This review is carried out by following the PRISMA guidelines and focusing mainly on the effects of bioactive compounds on oral stem cell-mediated dental, bone, and neural regeneration. It is observed that in recent years studies were mainly focused on the utilization of oral stem cell-mediated regeneration of bone or dental mesenchymal cells, however, the utility of bioactive compounds on oral stem cell-mediated regeneration requires additional assessment beyond in vitro and in vivo studies, and requires more randomized clinical trials and case studies.

## 1. Introduction

The term dental pulp stem cells was given by Gronthos to stem cells isolated from the dental pulp tissue of humans [1]. After the discovery of dental pulp stem cells (DPSCs) the researcher shifted their focus to other dental tissues for exploring new dental mesenchymal stem cell-like populations that resulted in the subsequent identification and characterization of stem cells, such as mesenchymal stem cells derived from gingiva (GMSCs) [2], stem cells from human exfoliated deciduous teeth (SHEDs) [3], stem cells derived from periodontal ligament (PDLSCs) [4], apical papilla derived stem cells (SCAPs) [5], and dental follicle precursor cells (DFPCs) [6]. Among different mesenchymal stem cell-like populations derived from human pulp tissue, SHEDs and DPSCs are more preferred as cell sources for regenerative medicine due to their easy accessibility and robust proliferative activity.

Initial demonstration of DPSCs’ robust proliferative activity was presented by Gronthos and coworkers in his study on human DPSCs (hDPSCs) derived from a single colony where the proliferation potential of human DPSCs was beyond 20 population doublings [7]. Mokry and coworkers’ work on hDPSCs lines further corroborated these findings, as proliferation potential beyond Hayflick’s limit was observed in all 11 lines studied with 81 being the highest population doubling number, indicative of their high potential for self-renewal [8]. Two different studies on a comparative evaluation of cell proliferation and colony-forming efficiency of hDPSCs and mesenchymal stem cells derived from human bone marrow (BMMSCs) have been reported. In the first study, cell proliferation and colony-forming efficiency of hDPSCs and BMMSCs from the same age group donors i.e., 18–25 years were studied [9], while in the second study the age group of donors was 19–29 years and 20–35 years for hDPSCs and BMMSCs, respectively [1]. Similar findings were reported in both studies that the cell proliferation and colony-forming potential of DPSCs was higher as compared to BMMSCs [1,9]. Cellular senescence and proliferation arrest are triggered by repeated cell divisions that induce a significant level of telomeres shortening, however, in the case of stem cells, telomerase shortening gets compensated by the stem cells [10].

Higher levels of telomerase expression in human DPSCs as compared to human BMMSCs have been reported, which would result in maintaining greater telomerase length in human DPSCs, probably accounting for their higher self-renewal activity and making them a suitable candidate for clinical use in regenerative therapy [5]. Due to the multilineage differentiation potential of DPSCs, they are capable of differentiating into cells isolated from all three germ layers i.e., mesoderm, endoderm, and ectoderm, making them a promising candidate for regenerative therapies in several diseases [10,11]. Because of the low immunogenicity and immunomodulatory properties of DPSCs, they are a suitable alternative for heterologous as well as autologous grafts [12,13,14]. Numerous studies have reported the regenerative ability of DPSCs in regenerating or repairing various somatic disorders such as muscular dystrophy [15], spinal cord injury [16], cerebral ischemia [17], cornea trauma [18], osteoporosis [19], acute myocardial infarction [20], diabetic neuropathy [21], glaucoma [22], and liver fibrosis [23].

The regenerative potential of stem cells isolated from the periodontal ligament (PDLSCs), a highly vascularized connective tissue located between the cementum of the root and alveolar bone socket wall, has been highlighted in several studies [24,25,26,27]. PDLSCs have been reported to exhibit characteristics similar to BMMSCs, having the potential to differentiate into chondrocytes, osteoblasts, and adipocytes under suitable differentiation conditions [24]. Their periodontal tissue regeneration potential was confirmed in animal models of periodontal defects, as they were able to form periodontal ligament structures and cementum [24,25,26,27].

Initially discovered in the remnant of dental pulp tissue of human exfoliated deciduous teeth, SHEDs exhibit characteristics similar to mesenchymal stem cells, such as their multi-differentiation potential, clonogenicity, cell proliferation capacity, fibroblastic features, and cell surface antigen expression [3,28,29]. The regenerative potential of SHEDs has been reported in systemic lupus erythematosus [30], spinal cord injury [31], liver fibrosis [28,32], hypoxic-ischemic brain injury [33], and ulcerative colitis [34].

The apical papilla of the tooth is a rich source of stem cells, often referred to as SCAPs, which can be easily obtained from immature teeth of adults [24]. SCAPs are known to have immunomodulatory properties and for having the ability to secrete a wide range of neurotrophic and regenerative growth factors [35]. Differentiation of SCAPs into chondrogenic, osteogenic, adipogenic, neurogenic, hepatogenic, and odontogenic lineages of cells have been reported in stem cell-based regenerative therapies in serval studies [6,36,37,38,39].

The lamina propria of the gingival connective tissue is a source of GMSCs, and they can be easily isolated from free and attached gingiva, hyperplastic gingiva, and inflamed gingival tissues [2]. The ability of GMSCs for multilineage differentiation has been highlighted in several studies, for having the potential to grow into osteocytes, keratinocytes, chondrocytes, odontogenic cells, endothelial cells, adipocytes, and neural cells [40]. GMSCs have gained significant attention in recent times as their availability and accessibility in gingival tissues make them an attractive alternative to other dental-derived mesenchymal stem cells for regenerative therapeutic interventions. The utility of GMSCs for potential regenerative applications such as sciatic and facial nerve regeneration [41,42,43], submandibular salivary glands regeneration [44], muscle regeneration [45,46,47], and bone and cartilage regeneration [48,49,50,51] has already been demonstrated, while it has shown promising therapeutic potential in periodontal diseases treatment [52], spinal cord injury [53,54], Psoriasis [55,56], Colitis [57,58,59,60], Arthritis [61,62,63,64], Lupus Nephritis [65], Osteoporosis [66], Atherosclerosis [67], Peri-Implantitis [68], Calvarial Bone Defects [69,70,71], Maxillofacial Bone Defects [72,73,74], Palatal/Gingival Defects [75,76,77,78], Oral Mucositis [79].

Oral stem cells of mesenchymal origin are reported to have similar properties comparable to mesenchymal stem cells derived from bone marrow. Despite being the preferred choice for replacement of the injured tissues and cells, the potential utilization of mesenchymal stem cells is limited in both syngeneic and allogeneic transplantations, as oxidative and inflammatory stress at the injured sites results in their weak survival rates after transplantation [80,81,82]. The crucial role of oxidative stress in determining the success of MSCs transplantation can be highlighted by the fact that uncontrollable amounts of reactive oxygen species (ROS) have been reported to change the in vitro differentiation pattern of MSCs into all three osteocytes, adipocytes, and chondrocytes cell types, by specific mechanisms. This can be better explained by an example, ROS-mediated under-expression of osteocalcin (BGLAP), OPN, CBF-α1 or *CBFA1*, or blockage of Wnt/β-catenin pathway appear to be responsible for the inefficiency of the osteogenic differentiation. The inefficiency of osteogenic differentiation is accompanied by an unwanted adipogenic differentiation, perhaps through ROS-mediated overexpression of PPARγ and C/EBPα (as the two adipogenesis-specific transcription factors), *FOXO* gene (as an osteogenesis attenuator), or reinforcement of the mTOR, NOX-4, and FOXO signaling pathways. As an antioxidant, *LCN2* can compensate for the inefficiency of osteogenic differentiation by stimulating the expression of *CBFA1*, which is a specific osteogenesis transcription factor. Further, the reactive oxygen species provided by NADPH oxidase 2 and 4 are needed for chondrogenesis differentiation. High levels of oxidative stress can also affect the telomere shortening rate, which results in senescence and apoptosis. In fact, following DNA damage, MSCs are arrested in either G1 to S or G2 to M phases, resulting in unwanted senescence [80,83]. Shaban et al., in their study, demonstrated that supplementation with antioxidants may not only be a viable option for enhancing cell survival, but also their differentiation potential [83]. The potential of Curcumin, a herbal metabolite in regenerative medicine, has been highlighted in several studies due to its ability to protect stem cells from oxidative stress damage, increase the proliferation and differentiation potential of stem cells, and postpone the aging process [84,85,86]. In vitro studies on DPSCs differentiation revealed that hypoxia and TNF-α synergistically inhibit the survival and osteogenesis of DPSCs. DPSCs, when supplemented with a 10 μM concentration of taxifolin, a flavonoid with significant antioxidant and anti-inflammatory effects, could substantially reduce the apoptosis of DPSCs by significantly increasing carbonic anhydrase IX (CA9) expression [87]. Several in vitro and in vivo studies have highlighted the role of Taxifolin in providing protection against oxidative stress-induced apoptosis and promoting osteoclastogenesis [87,88,89]. Artemisinin, a bioactive compound isolated from *Artemisia annua,* is reported to be useful in the treatment of malaria and colon cancer [90]. Artemisinin, at the dose of 40 μM, was able to reverse cell survival suppression caused by inflammation or hypoxia, along with reduced apoptotic rates and the expressions of pro-apoptotic proteins. Artemisinin was also successful in restoring osteogenic differentiation of DPSCs via upregulating the expression of CA9 and CA9-mediated antioxidant responses. This study also revealed that additional exposure to artemisinin could reactivate the Wnt/β-catenin signaling pathway in DPSCs in hypoxia or inflammation conditions, thereby promoting osteogenic differentiation of DPSCs [91].

The current study reviews oral stem cell-meditated dental, neural, bone, and other tissue regeneration and the role of bioactive compounds in oral stem cell-meditated regeneration. Only in vivo, clinical, and in vitro studies were included in the review, however, earlier many systematic reviews were published evaluating the utility of oral stem cells to differentiate into non-dental tissues, but none focused on oral stem cell-meditated dental, neural, bone and other tissue regeneration and utilization of various bioactive compounds in oral stem cell-mediated regeneration.

## 2. Research Methodology

The current study mainly focuses on the role of various bioactive compounds in oral stem cell-mediated regeneration, which is reviewed by following PRISMA 2020 guidelines ‘Preferred Reporting Items for Systematic Reviews and Meta Analyses’ [92]. Different studies were selected to be reviewed based on the eligibility criteria, including exclusion and inclusion criteria. Exclusion criteria: (i) clinical trials, in vivo and in vitro studies that do not follow ethical guidelines; (ii) studies not having full text accessible; (iii) studies published in native languages other than English.

Inclusion criteria: (i) clinical trials, in vivo and in vitro studies evaluating oral stem cell-mediated regeneration; (ii) findings published in the English language; (iii) recent studies published within the last 10 years (2010 to 2021) were reviewed; (iv) studies evaluating the effect of various bioactive compounds in oral stem cell-mediated regeneration.

For the literature search, electronic databases including Google Scholar, Scopus, Clinicaltrials.gov, Elsevier, and PubMed were used with the following keywords alone or in combination: in vitro, clinical trial, in vivo, oral stem cell, dental pulp, periodontal, mesenchymal, cell proliferation, osteoblasts, and bioactive compounds. The literature search was conducted within the period of 18 May 2021 to 12 December 2021 and studies published between 2010 to 2021 were reviewed. A database search showed a total of 165 studies, out of which, using the exclusion criteria, 8 studies were found to be duplicates, 10 studies does not have full text, 7 studies were published in local languages except English, and 4 in vivo, clinical trial and in vitro studies not following ethical guidelines were excluded, a further 136 studies were selected for review.

**Figure 1:**
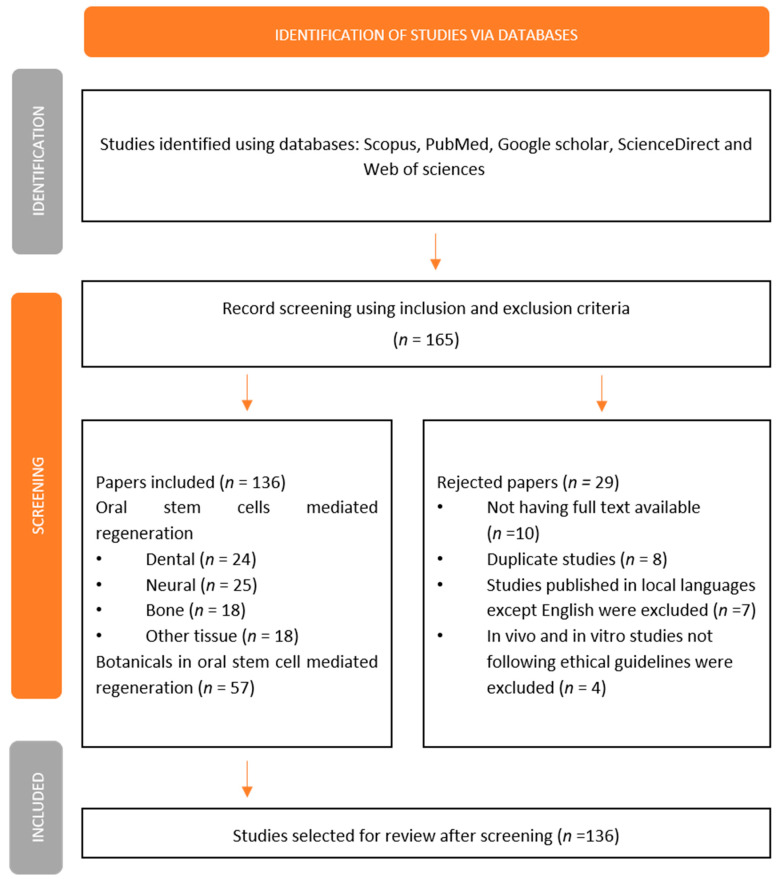
Flow chart showing selection process, following PRISMA 2020 guidelines.

From selected clinical trials, in vivo and in vitro studies, the following information is collected: compounds having potential bioactivities in oral stem cell-mediated dental regeneration, neural regeneration mediated by oral stem cells and repair, and oral stem cell-mediated bone regeneration. The flow diagram demonstrates the selection process, which includes the eligibility criteria: exclusion and inclusion, the number of studies found, and the number of studies reviewed in Figure 1.

## 3. Oral Stem Cell-Mediated Dental Regeneration

Recent advances in stem cell-mediated regeneration have shifted the focus of clinical dentists to regenerative endodontics, an alternative approach for the regeneration of dental tissues via stem cells, indicating a paradigm shift from artificial to biological replacement. The potential of DPSCs as seed cells for periodontal tissue regeneration, dentin pulp, and bone is being widely studied these days [93,94,95,96,97]. In a recent study, the formation of rich blood vessels in pulp-like tissues was observed within 6 weeks of implantation of a 3-dimensional (3D) scaffold-free dental pulp stem cells construct in the human root canal [98]. Histologic analyses in this study confirmed positive endothelial cells (human CD31) present at the center of regenerated tissue [98]. This study demonstrated the potential of pulpal tissue regeneration by DPSCs constructed in the pulpless tooth [98].

A number of studies have confirmed that DPSCs’ subcutaneous transplantation mixed with nanofibrous poly-L-lactic acid or tricalcium phosphate/hydroxyapatite could differentiate into odontoblasts and form dentin, including vascularized pulp-like tissue in immunodeficient mice [1,99,100,101,102]. Similarly, subcutaneous transplantation of SHEDs, with collagen type 1, hydrogel, or PLLA, resulted in the formation of pulp-like tissues, including odontoblasts and blood vessels in immunocompromised mice [103,104,105]. In an in vivo study on a canine periodontitis model, regeneration of periodontal ligament tissue and cementum was observed post-transplantation of DPSCs with bone granules into periodontal defects [106].

In another study, subcutaneous transplantation of DPSCs mixed with human salivary gland cells in an immunocompromised mouse resulted in enhancing the differentiation potential of human salivary gland cells to convert into functional salivary gland tissue [107]. In a study on pulp necrosis in human patients, DPSC implantation resulted in the regeneration of complete three-dimensional dental pulp tissue equipped with blood vessels and sensory nerves post 12 months of DPSC implantation [108].

Similarly, a pilot clinical study on irreversible pulpitis transplantation of mobilized DPSCs, along with atelocollagen granulocyte colony-stimulating factor into pulpectomized teeth, resulted in the formation of functional dentin in three out of the five patients studied [109]. A comparative study, analyzing the regenerative potential of DPSCs/HGF (hepatocyte growth factor)—DPSC injections and DPSC/HGF-DPSC sheets in repairing/regenerating periodontal bony defects in the upper and lower first molars of miniature pigs, revealed that periodontal regeneration/bone formation after grafting DPSC/HGF-DPSC sheets were higher after 12 weeks, compared to DPSC/HGF-DPSC injections [110]. Hu and coworkers findings on a similar model further corroborated the finding of Cao et.al that bone regeneration volume of 52.7 ± 4.1 mm^3^ in the case of DPSC sheets was significantly higher compared to the DPSC injections group, where bone regeneration volume was 32.4 ± 5.1 mm^3^ [110,111].

Similarly, in another study utilizing minipigs as a model system, PDLSCs, when seeded in Nanohydroxyapatite/chitosan/gelatin (nHA/CG) three-dimensional porous scaffolds, were able to significantly regenerate large bones with normal architectures and vascularization in jawbone defects within 12 weeks of implantation [112]. Several studies, which include pilot studies, pre-clinical studies, as well as clinical trials conducted on different in vivo models, have highlighted the regenerative potential of PDLSCs in periodontal complex regeneration, along with confirming the safety aspect of their use for therapeutic interventions in humans [113,114,115,116]. Recent studies conducted on xenogenic and autologous models have demonstrated the potential of Titanium implants with PDLSC sheets on the surface to stimulate the formation of periodontal ligament-like tissues and cementum-like tissues [117,118].

In recent preclinical studies, Enukashvily and coworkers demonstrated the efficiency of a unique approach in regenerative dentistry, utilizing an anatomical prototype-a mold representing the similar size and shape as the bone defects formed by 3D printing, and this 3-dimensionally printed form was then filled with a DPSC suspension and fibrin glue for repairing bone defects in a mice model. It was observed that the viability, immunophenotype, and osteogenic potential of DPSCs was maintained when DPSCs embedded in the fibrin glue were utilized for repairing bone defects. Another advantage of this approach was that apart from obtaining cell-containing implants similar to bone defects, it also allows cell migration and proliferation, and is hard enough to maintain its shape. Another advantage of this 3D-printed form for molding implants approach was that it could be utilized in implant formation with components that were usually not suitable for 3D printing. This technology could be utilized for the repair of bone tissue in dental medicine and maxilla-facial surgery [119]. In a minipig model of periodontitis, tissue defects were effectively restored 12 weeks after minipigs were subjected to local injection of SCAPs [120].

In a first of a kind study, PDLSCs from the same vial were differentiated into all three lineages i.e., fibrogenic, cementogenic, and osteogenic. Differentiation of PDLSCs in the suitable fibrogenic medium resulted in high expressions of periodontal ligament fibrogenic genes, such as elastin, *FSP-1*, *COL1*, and *COL3* at 28 days, with improvement in expression levels up to 20–70 fold, as compared to controls. Similarly, high expression of cementum genes (*CEMP1*, *CAP*, and *BSP*) and osteogenic genes (*ALP*, *RUNX2*, *OPN*) in PDLSCs was observed upon differentiation in a suitable cementogenic or osteogenic medium respectively. This study confirmed the differentiation of PDLSCs into fibrinogen, cementum, and bone-forming cells, thereby highlighting its potential for periodontium regeneration to form the bone-periodontal ligament–cementum complex [121].

The therapeutic potential of GMSCs in the periodontitis treatment was demonstrated in a study conducted by Liu and coworkers on the periodontitis model created in mice that were apolipoprotein E-deficient (ApoE^−/−^) with hyperlipidemia. In the study, it was observed that systematic transplantation of GMSCs in the tail vein in mice could significantly attenuate hyperlipidemia and inflammatory responses after nine weeks of transplantation, as demonstrated by a significant downregulation in mRNA expression levels of triglyceride (TG), interleukin (IL)-6, low-density lipoprotein cholesterol (LDL), sterol regulatory element binding protein-1c (SREBP-1c), alveolar bone loss (ABL) and total cholesterol (TC), whereas a significant upregulation was observed in the expression level of peroxisome proliferator-activated receptor α (PPARα), high-density lipoprotein cholesterol (HDL), and IL-10, as compared to control groups. On analysis of interradicular region histological changes, it was revealed that during weeks 1 and 2 post-transplantation, both control and GMSC-treated groups had deep periodontal pockets, attachment loss, inflammatory cell infiltration, and severe alveolar bone destruction, as confirmed by hematoxylin and eosin staining. However, this situation was reversed to a significant extent in the GMSC-treated group 4 weeks after transplantation, with lesser attachment loss, higher alveolar bone heights, periodontal pocket depth, and inflammatory cell infiltration. These findings highlight the therapeutic potential of GMSCs in improving periodontitis pathological conditions and promoting alveolar bone regeneration [52].

**Figure 2:**
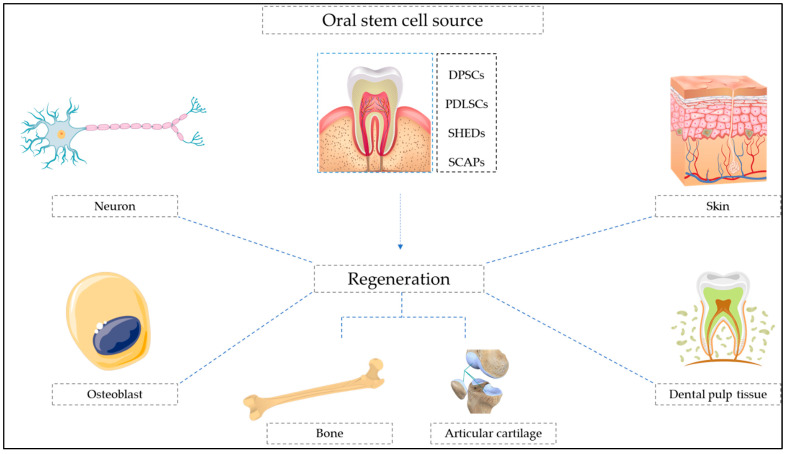
Diagrammatic representation of oral stem cell-mediated regeneration.

Figure 2 shows a diagrammatic representation of different types of oral stem cell-mediated regeneration.

## 4. Oral Stem Cell-Mediated Neural Regeneration and Repair

In an alternative strategy to the use of Schwann cell transplantation and autogenous nerve grafting for treating large gaps of peripheral nerve developed during peripheral nerve injuries, a nerve regeneration conduit was developed by Luo and coworkers. For the development of the nerve regeneration conduit (third generation), a combination of DPSCs, 10% gelatin meth-acryloyl, and human basic fibroblast growth factor (recombinant) was filled in a composite membrane tube of cellulose/soy protein. In vivo study on a rat model confirmed the potential of the developed nerve regeneration conduit in regenerating nerve tissues, such as myelinated nerve fibers, neurons, and Schwann-like nerve cells, and thereby successfully repairing a sciatic nerve with 15-mm long defect. The regeneration of nerve tissues at the damage site was a result of the direct differentiation of exogenous DPSCs, thereby highlighting that the therapeutic ability of DPSCs was on par with the conventional nerve autograft in the treatment of peripheral nerve injuries [122].

In a study carried out by Zhang and coworkers, DPSCs when subjected to neuronal inductive stimuli, acquired phenotype-resembling mature neurons, indicating their ability for neuron differentiation and neurosphere formation, as compared to other stem cells [123]. In this study, Zhang and coworkers also investigated the potential of DPSCs for stroke therapy by administering DPSCs in a rat model with focal cerebral ischemia, administration of DPSCs resulted in neuron-like cell trans-differentiation and reduction, and migration of cerebral edema (infarct size) in the brain [123]. An in vitro study on DPSCs revealed that after 72 h of treatment with base fibroblastic growth factor and epidermal growth factor, levels of the neuroprogenitor markers of sex-determining region Y-box 2 and nestin significantly increased in DPSCs. In the presence of neuron-like cells with a substantial rise in microtubule-associated protein 2 (MAP2), nestin and Neurogenin 1 (Ngn1) transcript levels were observed, confirming the potential of DPSCs to regenerate into neuron-like cells in suitable growth conditions [124].

In an in vivo study on male Sprague-Dawley rats modeling acute cerebral ischemia, it was observed that DPSC transplant not only successfully improved functional recovery of brain damage but also inhibited pro-inflammatory cytokine expression and microglial activation. Neuronal degeneration in the cortical ischemic boundary area was also attenuated by DPSC transplantation [125].

Interspinal transplantation of SHED in a rat model with spinal cord injuries resulted in significant hind limb functional recovery, as analyzed on the Basso, Beatie, and Bresnahan scale (BBB) Locomotor Rating Scale. The improvement in hindlimb movements of SHED rats was observed post one week of transplantation and remained substantial until the 6th week [31,126]. Histological evaluation conducted after six weeks of spinal cord lesion in control and SHED rats revealed that in the ventral horn of the spinal cord, SHED treatment partially preserved a number of motor neurons that accounted for the functional recovery in the BBB Locomotor Rating Scale [31].

These findings also revealed that a variety of events may contribute to the neuroprotective role of SHED, such as a reduction in the overexpression of pro-apoptotic factor neuronal nitric oxide synthase (nNOS), Tumor necrosis factor-α (TNF-α), and neuronal excitatory amino acid transporter 3 (EAAT3), maintained basal levels of the anti-apoptotic factor BCL-XL, contributing to less neuronal apoptosis, thereby confirming the neuroprotective role of SHED [31].

The ability of PDLSCs to survive, migrate, and undergo differentiation to neural cell-like phenotype, post-transplantation into the brain of 8-week-old immune-suppressed mice has been reported [127]. In a recent study on rodent brain cells, PDLSCs, when supplemented with appropriate growth media conditions, exhibited similar nuclear and cellular morphology to rodent brain cells. Development of Domains similar to dendrites and axon branches were also observed in this study and this was further validated by positive staining for F-actin and β-tubulin III. The results of this study also demonstrated that not only were morphological similarities present in these cells, but they could also communicate to each other via synaptic-like interactions and expressed related proteins, such as synapsin-1 and synaptophysin [128].

In the case of spinal cord injury (SCI), the neuro regenerative ability of DPSCs can be used to target multiple aspects of recovery, for example, it can inhibit the apoptosis of oligodendrocytes, neurons, and astrocytes induced by SCI, ultimately resulting in the improved neuronal filaments and myelin sheath preservation. Secondly, DPSCs can directly inhibit multiple axon growth inhibitors via paracrine mechanisms. Thirdly, lost cells were replaced by DPSCs differentiating into mature oligodendrocytes [129]. In a Wistar rat model of SCI, the neuroprotective role of SHEDs was highlighted, as it could reduce glial scar, cystic cavity area, and TNF-α levels, and increase neuronal filaments, and also resulted in the improvement of motor function [130].

In an AD cellular model, gradual restoration with re-elongation of retracted dendrites was observed in the cytomorphology of the DPSC-treated cells. Treatment also restored the neuron morphology, with elongated dendrites, thickened microtubular fibrils, and densely arranged microfilaments. Treatment also resulted in significant suppression of Tau protein phosphorylation at Ser 396 [131]. In vitro experiments showed that DPSC-derived Schwann cells can guide axonal extension and myelination [132,133]. Studies suggested that DPSCs can create a suitable microenvironment for neurite outgrowth and reinnervation on different types of neural lesions [134], even in retinal degeneration treatment [135]. Amelioration of dopaminergic (DA) neuron loss and behavioral deficits was achieved by the intrathecal graft of DPSCs in Parkinson’s disease murine models via upregulation of anti-inflammatory cytokines, including IL4, TNF-β, and IL2, and reduction of proinflammatory cytokines such as IL- 1β, IL6, IL-1α and IL8 [136].

Intravenous injection of SHED-derived conditioned medium at a concentration of 100 μg/mL exhibited improvement of motor deficits in the rotenone-induced Parkinson’s disease rat model [137]. Therapeutic effects of SHED-derived conditioned medium in the Parkinson’s disease rat model were mediated by amelioration of neuroinflammation, recovery of mitochondrial damage, and clearance of α-synuclein [137]. Grafting of DPSCs in a rat model with Huntington’s disease ameliorated striatal atrophy, repaired motor-skill impairment, and induced neurogenesis via the modulation of neuroinflammatory response and neurotrophic factors secretion [138]. In an in vitro model of Alzheimer’s disease, DPSC secretome degraded Aβ1-42 within 12 h of treatment via Aβ-degrading enzyme neprilysin [139]. SHEDs can act as a therapeutic intervention in Alzheimer’s disease in multiple ways, such as the promotion of neuroprotection, neurotransmission, axonal elongation, microglial regulation, and the suppression of inflammation. SHEDs on intranasal administration have been also reported to significantly improve cognitive function in mice models of AD [140]. Vasculogenesis and angiogenesis are essential components in CNS regeneration for providing oxygen and nutrients to spinal cord and injured brain tissue, and induction of both vasculogenesis and angiogenesis by DPSCs or their extracellular vesicles has been reported by several researchers [141,142,143,144]. Administration of DPSCs via intrahippocampal injection resulted in the generation of completely developed blood vessels, comprising perfectly aligned basement membranes, pericytes and endothelial cells in the rodent brain after one month of administration [141]. A conditioned medium of SHEDs could promote significant outgrowth of the dorsal root ganglion neurons, along with an increase in capillaries in mice models of diabetic polyneuropathy. It could also prevent the sensory nerve conduction velocities’ decline and with all factors, ultimately contribute to neural function improvement by SHEDs in diabetic polyneuropathy [145].

Zhang and coworkers demonstrated that even by utilizing non-genetic approaches, GMSCs of humans could be readily and reproducibly induced under suitable culture conditions into neural crest stem like cells (NCSC), with an improved expression of NCSC-related genes, such as *SOX9*, *NESTIN*, *TWIST1*, *p75NTR*, *SLUG*, *FOXD3*, and *SNAIL1* [42]. His study further confirmed the regenerative potential of NCSCs derived from GMSCs transplanted with collagen nerve conduits in a rat model with facial nerve transection, as continuity of nerve was observed post 12 weeks transplantation. After 4 to 12 weeks of transplantation, analysis of clinical nerve function was done which revealed that both GMSC-derived NCSCs group and GMSCs exhibited significant improvement in scores of facial palsy in comparison to nerve conduits only, with the best scores obtained in the GMSC-derived NCSCs group. Evaluation of facial nerve functional recovery, using the ratio of compound muscle action potential (CMAP) of the injured side in comparison to normal side at 12 weeks by electrophysiological analysis, revealed that the GMSC-derived NCSCs group exhibited twofold recovery of CMAP, as compared to the other two groups, thereby indicating the therapeutic potential of GMSC-derived NCSCs in the regeneration of facial nerve [42].

Zhang and coworkers, in their study on a rat model with sciatic crush injury, demonstrated that transplanted GMSCs at injury site regenerate into neuronal cells, although transplantation of GMSC-derived neural progenitor-like cells exhibited the potential to differentiate into Schwann cells as well as neuronal cells. The regenerative potential of GMSC-derived neural progenitor-like cells and GMSCs in promoting axonal regeneration at injury site and injured sciatic nerve distal segment was also confirmed in this study [146].

## 5. Oral Stem Cell-Mediated Bone Regeneration

DPSCs, when incorporated in polyglycolic acid (PGA) fiber scaffolds and transplanted into nude mice (8–12 weeks old) under mechanical loading conditions, were capable of forming mature tendon-like tissue after 14 weeks of implantation [147]. The DPSCs exhibited good osteogenic differentiation potential and were able to stimulate osteogenesis and bone regeneration in several in vitro as well as in vivo studies [148,149,150,151,152,153]. Implantation of human DPSCs cultured in an alginate scaffold significantly improved the cartilage regeneration in a rabbit model with cartilage damage 3 months post-implantation surgery [154]. These findings suggest the future utility of human DPSCs in articular cartilage regeneration and treatment of Osteoarthritis [154]. Similarly, the efficiency of nanocellulose–chitosan thermosensitive hydrogel embedded scaffolds with DPSCs for promoting cartilage formation was reported by both in vitro as well as in vivo approaches [155].

Therapeutic potential of DPSCs by skeletal muscle regeneration in animal models with peripheral nerve injury and Duchenne Muscular Dystrophy after achieving the commitment for Schwann and myoblast cells, respectively, have been reported in earlier findings [156,157]. Amelioration of ovariectomy (OVX)-induced osteopenia via reduction of T-helper 1 and Th17 cell numbers post-transplantation of SHEDs in the tail vein of OVX mice [30]. Transplantation of SHEDs results in T-cell apoptosis induction in OVX mice via activation of Fas pathway through Fas ligand (FasL), contributing to the downregulation of Th17 and Th1 cells and upregulation of regulatory T-cells (Tregs). Immunomodulation mediated by SHEDs rescues BMMSCs impairment induced by ovariectomy and osteoclastogenesis activation that results in increased bone mass [30].

In a recent study on a rat calvarial defect model, Huang and coworkers loaded PDLSCs in a specially designed recombinant human-like collagen, chitosan (CS) freeze-dried sponge (TRFS) and hybrid transforming growth factor-β3 (TGF-β3), to evaluate its potential for repairing calvarial bone defects. The results of this study demonstrated that PDLSCs successfully undergo osteogenic differentiation, which was further accelerated by the use of TGF-β3. This study highlights the future potential of PDLSCs as a therapeutic intervention for restoring traumatic defects of the skull [158].

In another study, Diomede and coworkers demonstrated the regenerative potential of GMSCs in bone tissue regeneration. In this study, GMSCs coupled with 3D engineered scaffolds (PLA) were utilized to regenerate bone tissue in vivo, as the use of PLA allows better mimicking of a micro-environment (endogenous) by providing 3D substrates that ultimately results in supporting cell differentiation, survival and proliferation. In this study, the efficiency of GMSCs, polyethyleneimine (PEI)-engineered extracellular vesicles (PEI-EVs), and extracellular vesicles (EVs) were also evaluated for bone regeneration. Morphological and transcriptomic analysis indicated that higher osteogenic inductivity was observed in the group with a combination of 3D-PLA + GMSCs + PEI-EVs. This combination was also successful in repairing the bone calvarial defect in a rat model with cortical calvaria bone tissue damage, as analyzed by computed tomography [70].

## 6. Oral Stem Cell-Mediated Regeneration of Other Tissues

Bosch and coworkers, in their study, demonstrated the potential utility of DPSCs as an autologous cell source for corneal endothelial therapies by adopting a two-step differentiation protocol for DPSCs. In the first step, DPSCs were differentiated into neural crest stem-like cells, confirmed by the overexpression of neural crest stem cell markers such as p75, Nestin, and AP2 markers, while in the second step these neurosphere/neural crest stem-like cells were then differentiated into corneal endothelial-like cells, confirmed by the higher expression level of markers such as of COL4A2, ZO-1, COL8A2, and ATP1A1 markers [14]. In vivo studies on a sodium iodate-induced model of retinal degeneration in Sprague-Dawley rats revealed that after 2 months of DPSCs transplantation, they effectively differentiated into different retinal cell types [117]. DPSCs were able to recover photoreceptor cells and retinal pigment epithelium, thereby restoring retinal morphology and visual functions to an extent in a rat model of retinal degeneration [159].

Intravitreally administration of DPSCs not only provided significant neuroprotection against retinal ganglion cell degeneration, but was also responsible for promoting optic nerve regeneration in the optic nerve crush injury model [160,161,162]. Similarly, a comparative study between BMMSCs and DPSCs revealed that DPSCs exhibited significantly more therapeutic efficacy in an experimental model of glaucomatous eye featuring cytokine-induced elevated eye pressure and associated RGC neurodegeneration, as compared to BMMSCs [22,162]. Successful corneal epithelium reconstruction in a rabbit model with limbal stem cell deficiency (LSCD) was reported post 3 months of transplantation of a cell sheet (tissue—engineered) composed of DPSCs into the injured cornea of the rabbit [163]. Human pluripotent stem cell differentiation into the retinal pigmented epithelium and retinal progenitor cells was promoted by SCAPs, likely via Wnt signaling pathway inhibition [164].

In a recent study, differentiation of DPSCs into cochlear hair cells was demonstrated, suggesting their potential as a regenerative therapy candidate for neural diseases such as sensorineural hearing loss (SNHL), which is primarily caused by dysfunction or death of cochlear cell types, due to their lack of regenerative capacity [165]. In another study, a rat model with stress urinary incontinence had human dental pulp stem cells (pre-differentiated) engrafted in the external urethral sphincter, which recovered its thickness, promoted vascularization and, exhibited significant recovery of urinary incontinence [93]. Apart from this, the presence of DPSCs within the nerve was indicative of their involvement in transected nerve repair [39]. Several studies have highlighted the potential of DPSCs to differentiate into smooth muscle cells when provided with suitable myogenic induction [166,167,168].

In an interesting in vitro study on the functional utility of cryopreserved stem cells for regenerative application, cryopreserved DPSCs with more than one year of cryopreservation were successfully differentiated into functional hepatocytes [169]. Transplantation of SHED-derived hepatocytes in rats led to the elimination of liver fibrosis and restoration of normal liver structure [170]. DPSCs trans-differentiation into esophageal stem cells was capable of repairing or regenerating damaged esophageal tissue in a rat model with radioactivity-induced esophageal injury [171]. Application of DPSCs in a femoral artery-ligated preclinical ischemic rat model restored limb functions, promoted muscle fiber regeneration, and increased angiogenesis, along with a reduction in inflammatory responses, indicating its potential utility as a therapeutic intervention for the treatment of critical limb ischemia [172]. Intravenously transplanted HGF-DPSCs in a rat model of ulcerative colitis could alleviate intestinal mucosa injuries via trans differentiation into intestinal stem cell-like cells, promotion of intestinal stem-cell like cell proliferation, reduction of oxidative stress, and suppression of inflammatory responses, suggesting its potential for clinical treatment of ulcerative colitis [173].

The role of SHEDs in hair regeneration was demonstrated in an in vivo study on a mice model, in which smearing the extract of SHEDs onto the C57BL/6 mice depilated dorsal skin and resulted in a lesser balder area, as compared to Minoxidil, an FDA-approved drug for hair loss treatment. This study revealed that SHED extracts were capable of hair regeneration boosting and shortening the cycle of hair regeneration via Glioma-associated oncogene 1 (*Gli1*) and Sonic Hedgehog (Shh) signaling pathway upregulation [174].

Table 1 shows relevant case studies of different oral stem cell-mediated neural, dental, bone and other tissue regeneration.

## 7. Botanicals in Oral Stem Cell-Mediated Regeneration

The interactions of natural plant extracts with stem cells have gained immense interest from researchers worldwide in recent times, as several studies have highlighted the ability of natural plant extracts/phytochemicals to promote survival, proliferation, as well as differentiation, of various mesenchymal stem cells [175,176,177,178,179,180,181,182,183,184]. In a recent study, curcumin, by increasing the early growth response protein 1 (EGR1) expression and activation of PI3K, Nrf2, and AKT signaling pathway, has been reported to promote the osteogenic differentiation potential in human PDLSCs [185,186]. In an in vitro study on PDLSCs’ osteogenic differentiation potential, it was revealed that advanced glycation end products resulted in attenuation of PDLSCs’ osteogenic differentiation ability via activation of canonical Wnt/β-catenin pathway; however, berberine hydrochloride, an isoquinoline alkaloid isolated from *Berberis vulgaris,* was able to reverse the PDLSCs’ osteogenic potential inhibition in an AGEs-enhanced microenvironment, partly by inhibition of the β-catenin and canonical Wnt pathway. Results show that berberine hydrochloride could be a potential therapeutic intervention for promoting PDLSCs’ osteogenic differentiation in diabetes-associated periodontitis patients [187].

Application of berberine along with SCAPs in a rat model with apical periodontitis in immature teeth, resulted in the formation of more tissues with longer roots, smaller apex diameters, and thicker root walls. SCAP osteogenesis was enhanced by berberine in a time and concentration dependent manner. Berberine was also found to be responsible for inducing the β-catenin expression and enhancing β-catenin entry in the nucleus, upregulating nuclear factor 2 downstream. Root repair enhancement in the case of immature teeth with apical periodontitis by the activation of β-catenin and canonical Wnt pathway in SCAPs was also reported in this study [188]. Similarly, berberine was also reported to increase the cell proliferation of DPSCs in a dose-dependent manner and promote dexamethasone-induced osteogenic differentiation via enhancement of Runx2 transcription factor activity that was followed by upregulation of osteogenesis marker expression. This study also confirmed EGFR and MAPK pathways’ role in promoting osteogenic differentiation of DPSCs by berberine, as both of the pathways were activated by berberine and inhibition of these pathways by inhibitors was responsible for significant suppression of the osteogenic differentiation promotion potential of berberine [189]. In addition to the role of Berberine in promoting osteogenic differentiation via different mechanisms in PDLSCs, SCAPs, and DPSCs in a dose dependent manner, the utility of antioxidant berberine has been widely reported for attenuating H_2_O_2_-induced apoptotic cell death of stem cells via quenching ROS production and increasing SOD activity. This study also revealed that Berberine, via upregulation of expression level of Bcl-2 and p-Akt, and downregulating the expression levels of Bax and cleaved caspase-3, could significantly reduce oxidative stress-induced apoptosis of stem cells [190]. The polyphenolic compound 2,3,5,4′-Tetrahydroxystilbene-2-O-β-D-glucoside (THSG) extracted from the dried tuber of *Reynoutria multiflora* (Thunb.) Moldenke, with strong antioxidant and free radical scavenging activities when supplemented in hDPSCs, could significantly enhance the renewal ability and proliferative potential of hDPSCs via the AMPK/ERK/SIRT1 axis [191].

The extracts of *Sapindus mukorossi* have been widely studied due to their wide range of pharmacological activities such as antioxidant, free radical scavenging, anti-inflammatory, anti-tumor, antifungal, and antimicrobial activities [178,192,193]. The seed oil of *Sapindus mukorossi* was reported to enhance the odontogenic/osteogenic differentiation potential of DPSCs by upregulation of ALP gene expression and mineralization-related extracellular vesicle secretion [175]. In an in vitro study on fraxinellone, commonly isolated from *Dictamnus dasycarpus,* it alleviated inflammation and promoted lipopolysaccharide-stimulated PDLSCs osteogenic differentiation via regulation of the BMP2/Smad pathway, thereby demonstrating its potential for clinical application in periodontitis treatment [194]. Rutin, a flavonoid with antioxidant and anti-free radical effects present in different fruits and vegetables, was responsible for the activation of AKT, mTOR, and PI3K signaling pathways via G protein-coupled receptor 30. AKT, mTOR, and PI3K signaling pathway activation by rutin resulted in a significant enhancement in PDLSCs’ potential for osteogenic proliferation and differentiation [195].

Apart from their role in PDLSC osteogenic differentiation and proliferation, rutin and resveratrol were reported to protect PDLSCs from the osteogenesis damage induced by TNF-α [196,197]. In recent in vitro and in vivo studies, the antioxidant and osteogenic potential of revestrol was demonstrated, with a concentration of 10 μM significantly increasing the cell viability of hDPSCs, via reduction in ROS activity, increasing the superoxide dismutase enzyme activity and glutathione concentration. The antioxidative and osteogenic potential was further confirmed by increased mRNA expression of Runx2, OCN, Sirt1, and Nrf2 genes [198]. This study also demonstrated that revestrol injected intraperitoneally in a Kunming mice model could significantly enhance the collagen and bone matrix formation, along with increased expression of Sirt1 gene [198]. Osthole, a coumarin derivative usually found in *Cnidium monnieri* with antioxidant properties, has been reported to effectively restore defects in osteogenic differentiation of PDLSCs via epigenetic modification. Osthole works by upregulating MOZ and MORF, histone acetylases that are responsible for specifically catalyzing the acetylation of histone3 lisine14 (H3K14) and histone3 lisine9 (H3K9), which act as a regulator for PDLSC osteogenic differentiation. This study also confirmed that Osthole with a 10−7 Mol/L concentration was best suited for the PDLSC proliferation and differentiation [199]. The antioxidant role of Osthole was highlighted in other study, as it could protect oxidative damage in stem cells via the PI3K/Akt-1 Pathway [200].

The utility of antioxidant naringin in promoting PDLSCs’ osteogenic differentiation and proliferation in both in vitro as well as in vivo models was reported, as it upregulates the expression levels of bone-related genes including COL1A2, OCN, RUNX2, and OPN at a concentration of 1 μM. Similarly, in an in vivo study on beige mice, the application of 1 μM naringin concentration resulted in the formation of a typical trabecular structure [201].

The odontoblastic differentiation of DPSCs was stimulated by shikonin via AKT–mTOR signaling pathway and CD44 antigen [202]. Isonymphaeol B, a prenylflavonoid isolated from *Macaranga tanarius* could stimulate the differentiation of DPSCs into odontoblasts, along with the formation of the tooth root and dentine via the MAP kinase and AKT signaling pathway. In vitro calcium mineral deposition was also enhanced upon application of Isonymphaeol B with DPSCs [203]. Modulation of Wnt/β-catenin signaling pathway and other key osteoblast-secreted proteins such collagen 1A1 and osteocalcin by ferutinin resulted in the activation and promotion of DPSCs’ osteogenic differentiation [204].

Concanavalin A (ConA) a lectin derived from the *Canavalia ensiformis* plant was reported to increase the osteogenic proliferation and differentiation potential of DPSCs, with 5 and 10 µg/mL of concentration. This study suggested that an increase in bone morphogenic protein-2 (BMP-2) by Concanavalin A can be attributed to the enhancement of osteogenic differential potential of DPSCs. The addition of Concanavalin A in DPSC cultures with a suitable osteogenic medium resulted in increased mineralization [205]. Purified ginsenoside Rg1 from stem or root of ginseng was reported to promote PDLSC and DPSC proliferation, PDLSC osteoblast differentiation, and odontoblast differentiation in DPSCs, thus making it a promising candidate for application in maxillofacial and oral regenerative medicine [206].

Ginsenoside Rg1 treatment promoted differentiation and proliferation of DPSCs via an increase in the expression level of genes, such as alkaline phosphatase (ALP), bone morphogenic protein-2, osteocalcin (OCN), fibroblast growth factor 2, and dentin sialophosphoprotein (DSPP) [207,208]. The osteogenic differentiation potential of PDLSCs is enhanced by ginsenoside Rg1 with 10 µmol/L of concentration, by enhancing the expression level of osteopontin (OPN), RUNX2, osteocalcin (OCN), collagen I, and ALP. However, at a concentration exceeding 100 μmol/L of ginsenoside Rg1, it was responsible for the inhibition of cell proliferation [209]. Wedelolactone, an antioxidant commonly occurring in *Wedelia calendulacea* and *Eclipta alba,* was capable of inducing significant odontoblast differentiation in DPSCs via neuropilin-1 (NRP1) and semaphorin 3A (Sema3A) pathway-mediated β-catenin activation, and inhibition of nuclear factor kappa B signaling pathway [210].

Application of acemannan, ‘a polysaccharide isolated from *Aloe vera’*, in the partial pulpotomy treatment revealed that it could stimulate DPSCs for the formation of predominantly normal pulp tissue organization and complete mineralized bridge covering the exposure site. In the case of teeth with reversible pulpitis, dentin regeneration was stimulated upon application of acemannan. This study also revealed that in vitro DPSC proliferation was significantly enhanced by acemannan, along with an increase in expression levels of type I collagen, BMP-2, vascular endothelial growth factor, BMP-4, alkaline phosphatase and dentin sialoprotein, and mineralization [211]. Epigallocatechin gallate (EGCG), which acts as an antibacterial crosslinking agent, resulted in the promotion of proliferation and differentiation potential of DPSCs in collagen scaffolds, suggesting its potential utility for regenerative endodontic therapy [212].

Asarylaldehyde treatment resulted in PDLSC osteogenic differentiation by increasing the mRNA expression level of osteoblast-specific markers in PDLSCs. Regulation of the transcriptional activity of alkaline phosphatase (ALP) via the p38/extracellular-signal-regulated kinase (ERK) signaling pathway was also observed upon asarylaldehyde treatment in PDLSCs [213]. Flavonoid kaempferol at a concentration of 10−6 M was reported to promote proliferation and mineral deposition of PDLSCs. It also upregulated expression levels of Bone Gamma-Carboxyglutamate Protein (BGLAP; osteocalcin), ALP, β-catenin, RUNX Family Transcription Factor 2, and Sp7 Transcription Factor (SP7; Osterix), with all factors contributing to the enhancement of proliferation and osteogenesis of PDLSCs, by activation of the β-catenin and Wnt signaling pathway [214]. The upregulation of early growth response gene-1 by Betulinic acid resulted in enhanced hPDLSCs osteogenic differentiation [215].

Flavonoid quercetin was able to attenuate the suppression of osteogenesis related genes, ALP and protein activity induced by TNF-α and mineralized matrix in PDLSCs via inhibition of NF κB/NLRP3 inflammasome pathway, thereby reversing TNF-α mediated osteogenic damage to PDLSCs [216]. Treatment of quercetin at concentrations of 2 and 5 μM significantly upregulated the antioxidant enzymes SOD1 and SOD2 in stem cells and increased the viability of cells [217]. The study also demonstrated the potential of quercetin in promoting osteogenic differentiation of stem cells via enhancing the phosphorylation of AMPK protein and upregulating the expression of the SIRT1 signaling pathway [217].

Methanolic extract of *Cirsium setidens* (Dunn) Nakai at a concentration of 0.05% significantly increased the viability of PDLSCs after 48 h of incubation. It was also responsible for enhancing the mineralization potential of PDLSCs. Enhancement of osteogenic potential in PDLSCs can be attributed to an increase in the expression level of alkaline phosphatase, collagen 1, runt-related transcription factor 2, and bone sialoprotein [218]. Salidroside isolated from *Rhodiola rosea* roots promoted DPSC cell viability, along with promoting their differentiation into odontogenic and osteogenic linage via activation of the BMP signaling pathway [219].

The flavonoid puerarin, with strong antioxidants in a concentration-dependent pattern, increased the activity of the ALP of PDLSCs, thereby enhancing the potential of osteogenic differentiation in PDLSCs [220]. Puerarin enhanced the osteogenic differentiation potential and viability of rat dental follicle stem cells via regulating the nitric oxide (NO) pathway [221]. The expression level of soluble guanylate cyclase (SGC), osteocalcin (OC), protein kinase G 1 (PKG-1), runt-related transcription factor 2 (RUNX2), collagen I, and osteopontin (OPN) was increased, along with an increase in activity of alkaline phosphatase (ALP), nitric oxide, and cyclic guanosine monophosphate upon treatment of puerarin with rat dental follicle stem cells [221].

PDLSCs’ osteogenic proliferation and differentiation was stimulated by Icariin in a dose dependent manner, having maximum stimulation at 0.1 μg/mL of concentration, however concentration of icariin above 10 μg · mL^−1^ proved to be cytotoxic [222,223]. In another study, application of icariin in a mouse model of calvarial defects and senescence increased trabecular bone mineral density and improved bone mass [223]. Treatment of PDLSCs with Moringin, an isothiocyanate obtained from *Moringa oleifera* seeds, resulted in the induction of PDLSC differentiation to neural progenitor cells via increased gene expression levels of genes that were involved in neuron cortical development, and specifically in neurons from the upper and deep cortical layers, as confirmed by next-generation transcriptomics sequencing. Apart from moringin’s role in neural progenitor cell differentiation of PDLSCs, it was also reported to upregulate genes involved in osteogenesis and adipogenesis, however to a much lower fold, as compared to neuronal differentiation [224].

Activation of sirtuin 1 (SIRT1) by resveratrol has been reported by several studies to induce bone marrow derived MSC structural morphological change and neuronal differentiation [225], dental pulp [226], cord blood [227], SCAPs [228], and umbilical cord [229] into the neural cells. Treatment of 15 μM resveratrol with DPSCs was capable of inducing NES gene expression, responsible for coding Nestin protein, that has been reported for their higher expression level of progenitor neural cells present in the subventricular zone of the human brain [230]. Similarly, pre-treatment with resveratrol with SCAPs resulted in the induction of neural progenitor marker gene expression, ultimately enhancing the induction of neural progenitor-like cells via a synergistic mechanism when provided with a suitable neuronal induction medium [228]. The aqueous leaves extract of *Acacia nilotica* promoted chondrogenesis induction in DPSCs by upregulating the expression of various proteins in the cellular matrix, such as aggrecan, sox9, glycosaminoglycan (GAG), and collagen 2α1 (Col2α1) that are crucial for the formation of cartilage tissue [231].

Girija and coworkers, in their study on GMSCs, highlighted the potential of *Acalypha indica* in increasing the wound healing ability of GMSCs. It was observed that wound closure activity of GMSCs was increased up to 56.91 ± 1.21% in 24 h when GMSCs were treated with 25 µg/mL concentration of methanolic extract of *Acalypha indica,* while the percentage of wound closure was further enhanced to 89.23 ± 1.09 % post 48 h of treatment. After induction with Acalypha indica, the morphology of GMSCs changed to polygonal cobblestone, which is a characteristic feature of keratinocyte-like cells. The transdifferentiation of GMSCs into keratinocyte-like cells was accompanied by a multifold increase in the expression levels of cytokeratin 5, cytokeratin 10, cytokeratin 14, involucrin, and filaggrin, indicating the potential of *Acalypha indica* and GMSCs as a promising alternative strategy for skin tissue engineering [232].

Pretreatment of GMSCs with nanostructured liposomes enriched with moringin for therapeutic intervention in spinal cord injury has shown promising results in a study conducted by Mammana et. al. The findings of this study revealed that the group receiving GMSCs–Moringin showed significantly faster recovery of motor function from day 4 post injury in ICR (CD-1) mice models of spinal cord injury, while the recovery in the group receiving GMSC treatment was a bit slower i.e., 8th day post trauma, thereby demonstrating the role of moringin in enhancing the therapeutic efficacy of GMSCs [54].

Treatment of hDPSCs with antioxidant baicalein, a flavonoid isolated from *Scutellaria baicalensis,* resulted in the upregulation of angiogenic factors and increased in vitro capillary-like tube formation. The upregulation of bone morphogenetic protein (BMP)-2 mRNA and phosphorylation of Smad 1/5/8 and Wnt ligand mRNA, glycogen synthase kinase-3, and nuclear β-catenin by baicalein treatment results in the promotion of angiogenesis and odontoblastic differentiation in hDPSCs via activation of the BMP and Wnt/β-catenin signal pathway [233]. Another study on baicalein conducted by Tian and coworkers highlighted its role as an effective hydroxyl radical-scavenger in protecting bone marrow-derived mesenchymal stem cells from hydroxyl radical-mediated oxidative stress, probably via two different mechanisms i.e., hydroxyl radicals direct scavenging via electron transfer, direct scavenging of hydroxyl radicals, possibly through electron transfer, and indirect inhibition of hydroxyl radical generation via Fe^2+^ chelation through the 4-keto-5,6,7-trihydroxy groups [234].

Apigenin, a natural flavonoid with strong antioxidant, and anti-inflammatory properties, could upregulate several osteogenesis-related signaling molecules, such as osteocalcin (OCN), bone sialoprotein (BSP), runt-related transcription factor 2 (RUNX2), BMP2, BMP4, and BMP7 in hDPSCs post 14 days of treatment [235]. This study also confirmed the role of apigenin in facilitating dentin-bridge formation with few irregular tubules in mice pulpal cavities after 42 days of treatment with apigenin [235]. Tanshinone IIA is a diterpene quinone derived from *Salvia miltiorrhiza,* and has been reported to induce hPDLSC osteogenesis via the ERK1/2-Runx2 axis signaling pathway [236]. In another study on improving the odontogenic differentiation potential of hDPSCs, both bakuchiol, a meroterpene phenol of *Cullen corylifolium,* and *C. corylifolium* extract were able to significantly enhance the odontogenic differentiation potential of hDPSCs via the upregulation of several odontogenic differentiation marker genes, such as dentin matrix acidic phosphoprotein-1, alkaline phosphatase, osteocalcin, and Runt-related transcription factor 2 [237].

Table 2 shows relevant case studies on the role of bioactive compounds in management of oral stem cell-mediated regeneration.

## 8. Conclusions

In recent years, a paradigm shift from artificial to biological replacement, due to recent advances in stem cell-mediated regeneration, is observed as clinical dentists were now more focused on regenerative endodontics. It is observed that oral stem cells mediated dental regeneration, such as periodontal tissue, and dentin pulp-like complex, have gained immense interest from researchers worldwide in recent times. Further, addressing oral stem cell-mediated regeneration, several studies have highlighted the ability of bioactive compounds to promote proliferation, differentiation, and survival of various mesenchymal stem cells. It is observed that utilization of DPSCs, SCAPs, PDLSCs, and GMSCs in dental, neural, bone, and other tissue regeneration shows potential results in clinical trials, and in vitro and in vivo studies. Botanicals and bioactive compounds such as revestrol, berberine, quercetin, and osthole have been reported to prevent oxidative stress-mediated apoptosis in the stem cells via different antioxidant defense mechanisms. Apart from increasing the viability of oral stem cells in the regenerative application, botanicals were also capable of promoting the differentiation of oral stem cells to a specific lineage. For the feasibility of using bioactive compounds to promote oral stem cell-mediated regeneration, additional randomized clinical trials are required, as well as in vivo and case studies. Oral stem cells were reported to be utilized for dental, neural, bone, and other tissue regeneration in various in vivo studies and clinical trials, however further investigation is needed to make their use uniform and technology available to all.

## Figures and Tables

**Table 1:** Oral stem cell-mediated dental, neural, bone and other tissue regeneration.

Cell Source	Study Type	Major Findings	References
**Oral Stem Cells Mediated Dental Regeneration**			
DPSCs	In vivo study to access viability of DPSC constructs for dental pulp regeneration	DPSCs implanted in human tooth root canal were differentiated into odontoblast-like mineralizing cells and human CD31—positive endothelial cells were found at the center of regenerated tissue.	[98]
hPDLSCs	In vivo study to examine bone regeneration potential of hPDLSCs with nHA/CG scaffolds in critical sized jawbone defects in minipigs	hPDLSCs with nHA/CG scaffolds increased new bone formation and generated bones larger in size with normal vascularization and architectures. It is also observed that in the bone marrow formed in the hPDLSCs/nHA/CG group, runt-related transcription factor 2 (Runx2) was highly expressed.	[112]
DPSCs	Pre-clinical study to develop technology of DPSCs seeded fibrin gel implant formation, with the same size and shape as the bone defect at the site of implantation	In mice, DPSCs seeded fibrin gel implants increased the bone tissue vascularization and volume.	[119]
hPDLSCs	In vitro study to examine the hPDLSCs differentiation into cementogenic, osteogenic, and fibrogenic lineages for the cementum-PDL-bone periodontium regeneration	In osteogenic medium, hPDLSCs shows high expressions of osteogenic genes i.e., *ALP*, *RUNX2*, *COL1*, and *OPN* (14 and 21 days), produced ALP activity and mineral nodules (5 and 10 folds of control).	[121]
		In fibrogenic medium, hPDLSCs show increased PDL fibrogenic genes expression levels, including *FSP-1*, *PLAP-1*, *COL1*, elastin, and *COL3* (28 days) (20–70 folds of control).	
		In cementogenic medium, hPDLSCs showed high expressions of cementum genes i.e., *BSP*, *CEMP1*, and *CAP* (21 days) (10–15 folds of control), and synthesized mineralized cementum 40 folds via ALP staining and 50 folds via ABS.	
**Oral Stem Cell-mediated Neural Regeneration**			
DPSCs	To examine the efficiency of the combination of DPSCs with bioactive hydrogels for repairing large gap peripheral nerve injuries	Direct differentiation of exogeneous DPSCs in CSM-GF conduit resulted in the formation of new nerve tissues at the defect site. This study also demonstrated that bioactive hydrogels combined with DPSCs could regenerate myelinated nerve fibers and Schwann cells.	[122]
DPSCs	To examine the efficacy of DPSCs neuronal differentiation induction by EGF and bFGF.	EGF and bFGF-treated DPSCs shows increase in the expression of the neuroprogenitor markers of SRY (sex determining region Y)-box 2 (SOX2) and nestin after 72 h.	[124]
		A significant increase in transcript levels of nestin, neurogenin 1 (Ngn1), and microtubule-associated protein 2 (MAP2) was observed post treatment as compared to cells maintained in the control media. Treatment also resulted in formation of some neuron-like cells.	
SHEDs	To examine the efficacy of conditioned medium from SHEDs in improving cognitive function in Alzheimer’s disease mouse model	Pro-inflammatory responses induced by β-amyloid plaques is attenuated by SHED-CM and generated a tissue-regenerating/anti-inflammatory environment, accompanied by anti-inflammatory M2-like microglia induction.	[140]
SHEDs	In vivo study to examine the beneficial effects on diabetic polyneuropathy in mice by secreted factors in conditioned medium of SHED-CM	In the diabetic mice model, the decline in sensory nerve conduction velocities was significantly prevented in SHED-CM as compared to DMEM. Neurite outgrowth of dorsal root ganglion neurons was also significantly enhanced in SHED-CM.	[145]
**Oral Stem Cell-mediated Bone Regeneration**			
SHEDs	In vivo study to examine the one-time transplantation of SHED may prevent the tail vein ameliorates ovariectomy (OVX) -induced early osteoporotic phenotype	SHED via a FasL/Fas pathway mediated T-cell apoptosis which as result ameliorates the osteopenia phenotype and immune tolerance in OVX mice.	[30]
DPSCs	In vivo study to examine DPSCs for possible application in tendon tissue engineering	It is observed that when DPSCs was transplanted in aligned PGA fiber scaffolds, tendon-related markers including tenascin-C, scleraxis, collagens I, eye absent homologue 2 and VI and tenomodulin were significantly enhanced.	[147]
		DPSC-PGA constructs on transplantation in a mouse model resulted in the formation of mature tendon-like tissue under mechanical loading conditions.	
hDPSCs	A preliminary in vivo study to examine chondrogenic ability of hDPSCs (cultured in an alginate scaffold) to regenerate articular cartilage	It is observed that hDPSCs express collagen II and aggrecan. Significant cartilage regeneration was observed 3 months post implantation of hDPSCs cultured in 3% alginate hydrogels in a rabbit model with cartilage damage.	[154]
hPDLSCs	In vivo study to examine effects of hPDLSCs with RHC/CS scaffolds on the repair of critical-size skull injury in rats	It is observed that hPDLSCs proliferate and undergo osteogenic differentiation in TRFS (p < 0.05) accelerated by TGF-β3.	[158]
**Oral Stem Cells Mediated Regeneration of Other Tissues**			
DPSCs	In vitro study to examine the potential utility of DPSCs as an autologous cell source for corneal endothelial therapies by adopting a two-step differentiation protocol for DPSCs	DPSCs were differentiated into neural crest stem-like cells, confirmed by the overexpression of neural crest stem cell markers such as p75, nestin, and AP2 markers.	[14]
		In second step neural crest stem-like cells were then differentiated into corneal endothelial-like cells, confirmed by the higher expression level of markers such as of COL4A2, ZO-1, COL8A2, ATP1A1 markers.	
DPSCs	In vitro study to examine the ability of DPSCs to differentiate into cochlear hair cell	DPSCs were successfully able to differentiate into neural stem cells with mean 24% nestin-positive cells.	[165]
		NSCs derived from DPSC were differentiated into inner ear hair cell-like cells with 81% average cells presenting myosin VIIa.	
hDPSCs—cryo	In vitro study to examine ability of long-term cryopreserved dental pulp tissues to differentiation into HLCs and DE cells	It is observed that hDPSCs—cryo were successfully differentiated into DE and functional hepatocytes. Differentiated HLCs (30th day) and DE cells (6th day) significantly increased hepatocyte- and DE-specific markers at the mRNA and protein level.	[169]
SHED	In vivo study to examine ability of SHED with C57BL/6 mice skin cells to improve hair regeneration	It is observed that SHED up regulated the expression of Shh and Gli1 pathway.	[174]
		SHED and skin cells of C57 mice when co-transplanted to nude mice, they were found to promote hair regeneration.	

**Abbreviations**: DPSCs—Dental pulp stem cells; hPDLSCs—human periodontal ligament stem cells; GFD—10% GelMA hydrogel, recombinant human basic fibroblast growth factor and DPSCs; CSM—composite membrane; SHED—stem cells of human exfoliated deciduous teeth; DMEM—serum-free Dulbecco’s modified Eagle’s medium; PGA—polyglycolic acid; TGF—transforming growth factor; HLCs—hepatocyte like cells; DE—definitive endoderm.

**Table 2:** Role of bioactive compounds in management of oral stem cell-mediated regeneration.

Source	Bioactive Compounds	Type of Study	Major Findings and Mechanism of Action	References
*Artemisia annua*	Artemisinin (Sigma Aldrich, St. Louis, MO, USA)	In vitro study investigated the effect of Artemisinin on hypoxia and TNF-α mediated osteogenesis impairment in DPSCs	Artemisinin reversed the suppression of cell survival caused by hypoxia or inflammation in DPSCs, along with restoring the osteogenic differentiation potential of DPSCs	[91]
*Sapindus mukorossi*	Seed oil (He He Co., Ltd., Taipei, Taiwan)	In vitro study to examine the effects of *S. mukorossi* (seed oil) on the differentiation and proliferation of DPSCs	Enhanced the odontogenic/osteogenic differentiation potential of DPSCs by upregulation of ALP gene expression and mineralization-related extracellular vesicle secretion	[175]
*Curcuma longa*	Curcumin (Sigma Aldrich, St. Louis, MO, USA)	In vivo study on effect of curcumin on hPDLSCs osteogenic differentiation	Curcumin increased protein and mRNA levels of COL1, ALP, RUNX2, and activated PI3K/AKT/Nrf2 signaling pathway	[185]
*Curcuma longa*	Curcumin (Solarbio Life Sciences, China)	In vivo study Curcumin displays promoting osteogenic differentiation and its mechanism	Curcumin 10 µmol/L treatment maximal promoting the cells viability, ALP activities, mineralization, and levels of Runx2, OC, OPN, Collagen I, and EGR-1 in hPDLSCs	[186]
*Berberis vulgaris*	Berberine hydrochloride (Wako Pure Chemical Industries, Ltd., USA)	In vitro study to examine effects of AGE and berberine hydrochloride on the hPDLSCs’ osteogenic differentiation ability	Berberine hydrochloride was able to reverse the inhibition of the PDLSCs’ osteogenic potential in an AGEs enriched microenvironment, partly by inhibition of the β-catenin and canonical Wnt pathway	[187]
*Berberis vulgaris*	Berberine (Sigma Aldrich, St. Louis, MO, USA)	In vivo study to examine the effect of berberine on rat root canals of immature teeth with apical periodontitis	Berberine induced β-catenin expression and activated the β-catenin and canonical Wnt pathway in SCAPs which improved root repair in immature teeth with apical periodontitis.	[188]
*Berberis vulgaris*	Berberine (Sigma Aldrich, St. Louis, MO, USA)	In vitro study to examine effects of berberine on the osteogenesis and cell proliferation of DPSCs	Berberine enhanced hDPSC cell proliferation in a dose-dependent pattern and activated MAPK–EGFR–Runx2 signaling pathways.	[189]
*Reynoutria multiflora* (Thunb.) Moldenke	2,3,5,4′-Tetrahydroxystilbene-2-O-β-D–glucoside (THSG) (Taipei Medical University, Taipei, Taiwan)	In vitro study investigated the effect of THSG on cell proliferation in hDPSCs.	THSG treatment enhance d the renewal ability and proliferative potential of hDPSCs via the AMPK/ERK/SIRT1 axis	[191]
*Dictamnus dasycarpus*	Fraxinellone (Chengdu Herbpurify Co., Ltd., Chengdu, China)	In vitro and in vivo study to examine antitumor effects of fraxinellone on lung cancer cells	Fraxinellone treatment inhibits expression of PD-L1 by HIF-1α and STAT3 signaling pathway downregulation, further inhibiting angiogenesis and proliferation in cancer cells	[194]
*Fagopyrum esculentum*	Rutin (Solarbio Science & Technology Co., Ltd., Beijing, China)	In vitro study to examine the effects of rutin on the PDLSCs’ osteogenic proliferation and differentiation	Rutin increased osteogenic differentiation and proliferation of PDLSCs by GPR30-mediated PI3K/AKT/mTOR signal transduction	[195]
*Cnidium monnieri*	Osthole (National Institutes for Food and Drug Control, Beijing, China)	In vitro study to determine osthole efficiency against defective osteogenic differentiation of P-PDLSCs via epigenetic modification	Osthole (10^−7^ Mol/L) upregulated MORF, MOZ, and histone acetylases that catalyze acetylation of Histone 3 lisine14 (H3K14) and Histone 3 lisine9 (H3K9)	[199]
			Osthole treatment enhanced bone formation and cell sheet formation of PDLSC sheets in periodontitis (animal models)	
*Drynaria roosii* Nakaike	Naringin (National Institute for the Control of Pharmaceutical and Biological Products, China)	In vitro and in vivo study to examine the effect of naringin on proliferation and osteogenic differentiation of hPDLSCs	Naringin (1 µM) efficiently promoted hPDLSC differentiation and proliferation and increased expression levels was observed in related genes (COL1A2, OPN, RUNX2, and OCN) as compared to the control group	[201]
*Macaranga tanarius*	Isonymphaeol B (Okinawa, Japan)	In vitro study to identify odontogenesis-promoting compounds and examine the molecular mechanism underlying enhanced tooth formation and odontoblast differentiation	Isonymphaeol B shows stimulatory effects on tooth root, dentine formation and odontoblast differentiation via AKT and MAP kinase signaling pathways	[203]
*Canavalia ensiformis*	Concanavalin A (Sigma Aldrich, USA)	In vitro study to determine the effect of concanavalin A on osteogenic and proliferation differentiation of DPSCs	Concanavalin A at concentration of 5 and 10 µg/mL to DPSCs significantly increased the osteogenic and proliferation differentiation of DPSCs (*p* ≤ 0.05)	[205]
*Panax ginseng*	Ginsenoside Rg1 (Bio-function, Beijing, China)	In vitro study to examine the effects of ginsenoside Rg-1 on osteogenic differentiation and proliferation of hPDLSCs	Ginsenoside Rg-1 enhanced osteogenic differentiation and proliferation of hPDLSCs at an optimum concentration of 10 μmol/L	[209]
*Eclipta prostrata*	Wedelolactone (Dalian, China)	In vitro study investigated effect of wedelolactone on odontoblast differentiation of DPSCs	Wedelolactone induced odontoblast differentiation through NRP1, Sema3A, and NF-κB pathway inhibition and activation of b-catenin pathway	[210]
*Aloe barbadensis* Mill.	Acemannan (Chulalongkorn University, Bangkok, Thailand)	In vitro study to examine effects of acemannan on human deciduous pulp cells and the response after vital pulp therapy in dog deciduous teeth	DPSC proliferation was significantly enhanced by acemannan along with an increase in expression levels of type I collagen, BMP-2, vascular endothelial growth factor, BMP-4, alkaline phosphatase, dentin sialoprotein, and mineralization	[211]
*Cirsium setidens* (Dunn) Nakai	Methanolic extract (Kangwon National University, Republic of Korea)	In vitro study to examine the effects of methanolic extracts of *C. setidens* on osteogenic differentiation of hPDLSCs	Methanolic extract treatment with concentration of 0.05% significantly increased the viability of PDLSCs and also increased the expression levels of alkaline phosphatase, collagen 1, runt related transcription factor 2, and bone sialoprotein	[218]
*Rhodiola rosea*	Salidroside (Chengdu Must Bio-Technology Co., Shanghai, China)	In vitro study to investigate the effect of salidroside on the odontogenic differentiation and proliferation of hDPSCs	Treatment with salidroside promoted DPSCs cell viability, along with promoting their differentiation into odontogenic and osteogenic linage via activation of the BMP signaling pathway	[219]
*Moringa oleifera*	Moringin (Indena India Pvt. Ltd.; Bangalore, India)	In vitro study to examine efficacy of moringin to induce PDLSCs toward neural progenitor differentiation	Treatment of PDLSCs with moringin resulted in the induction of PDLSC differentiation to neural progenitor cells via increased gene expression levels of genes that were involved in neuron cortical development	[224]
*Acacia nilotica*	Aqueous leaves extract (Hormavu, Bangalore)	In vitro study to investigate the efficacy of Acacia nilotica leaves extract in chondrogenesis induction from mesenchymal stem cells	Treatment of aqueous leaves extract promoted chondrogenesis induction in DPSCs by upregulating the expression of various proteins in the cellular matrix, such as aggrecan, sox9, glycosaminoglycan (GAG), and collagen 2α1 (Col2α1)	[231]
*Acalypha indica*	Methanolic extract (Porur, Chennai, India)	In vitro study on GMSCs highlighted the potential of A. indica (methanolic extract) in increasing the wound healing ability of GMSCs	Treatment of *A. indica* extract (25 µg/mL) wound closure activity of GMSCs was increased up to 56.91 ± 1.21% in 24 h, while the percentage of wound closure was further enhanced to 89.23 ± 1.09% post 48 h of treatment	[232]
*Scutellaria baicalensis*	Baicalein (Sigma Aldrich, USA)	In vitro study investigated the effect of Baicalein on Angiogenesis and Odontoblastic Differentiation	Baicalein promoted odontoblastic differentiation and angiogenesis of HDPCs by activating the BMP and Wnt/β-catenin signal pathways	[233]
*Salvia miltiorrhiza*	Tanshinone IIA (Sigma Aldrich, USA)	TSA affects the osteogenic differentiation of hPDLSCs.	Tanshinone IIA can induce hPDLSC osteogenesis through the ERK1/2-Runx2 axis	[236]
*Cullen corylifolium* L.	Bakuchiol and *C. corylifolium* extract (KMD medicinal herbs Co., Yunnan, China)	In vitro study to examine the efficacy of Bakuchiol and *C. corylifolium* extract as differentiation-inducing substances for tooth regeneration, was determined by monitoring odontogenic differentiation in hDPSCs	Bakuchiol and *C. corylifolium* extract significantly enhance the odontogenic differentiation potential of hDPSCs via upregulation of odontogenic differentiation marker genes, such as dentin matrix acidic phospho-protein-1, alkaline phosphatase, osteocalcin, and Runt-related transcription factor 2	[237]

## Data Availability

Not applicable.
